# Exploring the Relevance of S100A8 and S100A9 Proteins in Preeclampsia: A Narrative Review

**DOI:** 10.3390/ijms262010118

**Published:** 2025-10-17

**Authors:** Melinda-Ildiko Mitranovici, Laura Caravia, Ioan Emilian Oală, Andreea Taisia Tiron, Corneliu-Florin Buicu, Dan Dumitrascu-Biris, Mihai Munteanu, Viviana Ivan, Adrian Apostol, Ion Petre, Lucian Pușcașiu

**Affiliations:** 1 Department of Obstetrics and Gynecology, Emergency County Hospital Hunedoara, 14 Victoriei Street, 331057 Hunedoara, Romania; oalaioanemilian@gmail.com; 2 Faculty of Medicine, “Carol Davila” University of Medicine and Pharmacy, 050474 Bucharest, Romania; taisia_andreea@yahoo.com; 3 Public Health and Management Department, “George Emil Palade” University of Medicine, Pharmacy, Sciences and Technology, 540142 Targu Mures, Romania; florin_buicu@yahoo.com (C.-F.B.); dr.dan.biris@gmail.com (D.D.-B.); puscasiu@gmail.com (L.P.); 4 Faculty of Electrical Engineering, Technical University, George Baritiu Street, 400394 Cluj Napoca, Romania; mihai.munteanu@ethm.utcluj.ro; 5 Department of Cardiology, “Victor Babes” University of Medicine and Pharmacy, 2 Eftimie Murgu Sq., 300041 Timisoara, Romania; ivan.viviana@umft.ro (V.I.); adrian.apostol@umft.ro (A.A.); petre.ion@umft.ro (I.P.)

**Keywords:** preeclampsia, placenta, S100A8, S100A9, calprotectin

## Abstract

Preeclampsia is a common pregnancy complication that may threaten the health of pregnant women and their fetuses. Intrauterine growth restriction is the most serious complication in fetal development observed in preeclampsia. In their later years, women with preeclampsia are at risk of cardiovascular disease. The use of multiple biomarkers can usually improve diagnostic performance. Currently, S100A8 and S100A9 are gaining increased attention as inflammatory markers of preeclampsia. Our review aims to present an integrated view of the potential role of S100A8/A9 in the physiopathology of preeclampsia. We also explored the potential role as biomarker or in targeted treatment. At the moment, there is no efficient treatment for PE other than birth. Understanding the pathological mechanisms and the importance of calprotectin can shape a new strategy for large-scale approaches to change diagnostic and therapeutic management. The value of the calcium binding proteins S100A8/A9 as a potential prognostic biomarker in placental disfunctions based on malperfusion has not yet been investigated. Encouraging results have been obtained in experimental and clinical interventional studies with S100A8/A9 blockers as a form of therapeutic management for autoimmune disease and cancer. It may also be limited because of safety issues for the fetus. However, future investigations can emerge.

## 1. Introduction

Preeclampsia (PE) complicates 5–8% of pregnancies. It is defined as elevated blood pressure (>140/90 mmHg on at least two occasions and >6 h apart) at or beyond 20 weeks of gestation and proteinuria > 300 mg/day [1,2,3]. Conventionally, PE was characterized by new onset hypertension and proteinuria, but this definition has been revised to include cases with different organs being affected, and with proteinuria no longer a requirement. Fetal growth restriction has also been considered as a marker of end organ involvement [4,5]

Two types of PE have been distinguished: early-onset, diagnosed before 34 weeks of gestation, and later-onset, after 34 weeks of pregnancy. The pathogenesis of both early- and late-onset PE is not fully understood. Histopathological examination of placentas shows infarcts and sclerotic arterioles [2]. Early-onset PE is often associated with (HELLP) syndrome (hemolysis, elevated liver enzymes, low platelet count) and intrauterine growth restriction (IUGR). Late-onset PE is more frequent (comprising more than 80% of all cases worldwide) and can be associated with hypertension, kidney disease, obesity, and diabetes but without intrauterine growth restriction [2]. Thrombocytopenia can occur (platelet count < 100,000/microl), along with renal failure, liver disfunction, and pulmonary and cerebral edema. Serious complications in fetal development have been observed in cases of PE, such as IUGR, premature birth, stillbirth, or fetal demise. Studies have shown that, in their later years, pregnant women with PE are at risk of cardiovascular disease [2,3,6]. Even though a great deal of research on gestational hypertension has been conducted to identify diagnostic and prognostic biomarkers, the pathological pathway has yet to be clearly determined. As a result, these biomarkers have not yet been fully identified.

The most common cause of gestational hypertensive disorders is vascular placental malperfusion due to the impaired remodeling of uterine spiral arteries [1,3], which leads to placental ischemia/hypoxia with the release of ROS and anti-angiogenic factors, such as soluble FMS-like tyrosine kinase 1 (sFlt1) and soluble endoglin (sEng), both involved in PE and produced in the placenta [7]. Of these, soluble FMS-like tyrosine kinase 1 (sFlt1) is the main antiangiogenic factor with a negative effect on the endothelium and, consequently, on the placenta. It has been shown to be the most important clinical marker for PE, used in the first trimester screening, along with pregnancy-associated placental protein- A (PAPP-A) and placental growth factor (PlGF), which decrease in PE [7,8,9].

At the moment, there is no efficient PE treatment other than birth, often through premature cesarean section, with the consequences of prematurity and hypoxia for neonates. The development of soluble biomarkers for early diagnosis and the discovery of potential new mechanisms that can be therapeutically targeted to prevent PE and its consequences are therefore of high clinical importance. We will try to understand the role of S100 proteins in this process.

S100 is a family of low-molecular-weight calcium-binding proteins (9–14 kDa) [10]. S100A8, known as calgranulin A, and S100A9, known as calgranulin B, are myeloid-related proteins (MRP) that belong to the S100 family and are involved in regulating pathological processes such as inflammation [11,12,13]. The heterodimer S100A8/S100A9, known as calprotectin, is involved in the immune system, but altered expression of S100 family members is also found in cancers [2]. S100A8/A9 is an endogenous ligand of Toll-like receptor 4 (TLR4) and of the receptor for advanced glycation end products (RAGE). It has been shown to promote atherogenesis in animal models, and in humans, it correlates with atherosclerosis. The S100A8/A9-TLR4 interaction has also been shown to be involved in the pathogenesis of systemic infections and autoimmune diseases [2,12,13,14,15,16,17,18].

Their exact biological role is under-investigated. Thus, S100A8/A9 might represent a useful plasmatic biomarker and therapeutic target in preeclampsia [13,15,19,20,21]. S100A8/A9 blockers have been developed, and their clinical testing is approved [13].

Our review aims to present an integrated view of the potential role of S100A8/S100A9 in the physiopathology of pre-eclampsia.

## 2. Data Search

This study was conducted in 2024, using distinct search mechanisms according to the PRISMA guidelines [22]. We took into consideration the last 15 years, using specific keywords such as pre-eclampsia, intrauterine growth restriction, inflammation, S100A8, S100A9, and calprotectin. An extensive literature review was conducted using Google Scholar and PubMed, generating 663 titles. Editorials, books, reports, or studies not aligned with our objectives were excluded. The inclusion criteria included full-text articles written in English with proper study design; in addition, clear and informative articles were sought. Other articles for which only the Abstract was available were removed, as were duplicates or studies with inappropriate designs.

**Figure 1:**
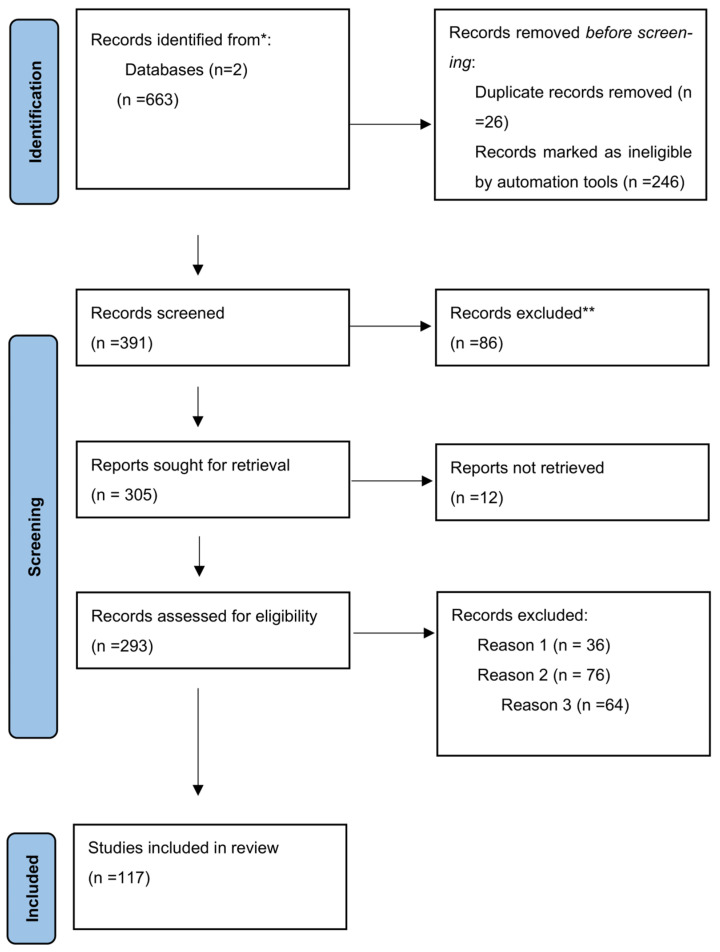
* The number of records identified from Google Schoolar and PubMed databases. ** Records excluded by an investigator. Reason 1: Records excluded publications in a language other than English; Reason 2: Records excluded by a human reviewer due to inaccurate or inappropriate titles; Reason 3: Records excluded based on the study’s research design.

The suitability of articles during the selection process was established by two authors, based on inclusion/exclusion criteria. We took into account articles that bring new knowledge regarding screening biomarkers in PE, with a prognostic role, or elements that are useful in targeted treatments based on molecular pathology. Ultimately, 117 articles were chosen. A narrative approach was adopted due to the characteristics of data pooling also better suited to addressing a topic in such wider ways. A rigorous evaluation for quality, reliability, and validity was performed. also better suited to addressing a topic in such wider ways. SANRA quality assessment scale was used, based on the relevance for the topic, the aim of the review, evidence level and relevant endpoints. [23] The selection method is described in the flow diagram (Figure 1).

## 3. General Functions of S100A8, S100A9, or Calprotectin

The primary function of the innate immune system, and of its central components, neutrophils and monocytes, is to combat pathogen invasion. However, it can also be activated by endogenous danger-associated molecular patterns (DAMPs), known also as alarmins, which are released after tissue damage under conditions of immunological stress [2,3,12,13]. S100A8/A9 is an alarmin produced by cells of myeloid origin [13]. Its signals can be activated through the Toll-like receptor 4 (TLR-4), and promote inflammation [13,24,25,26,27]. S100A8 and S100A9 can also be expressed upon activation of dendritic cells [10,28,29], macrophages, endothelial cells [29,30], fibroblasts [31], and keratinocytes [2,12,13,28,29,30,31,32]. S100A8/A9 have been studied not only in terms of correlations with the circulating numbers of neutrophils, but also with platelets in human blood. Both pro- and anti-inflammatory functions have been reported for S100A8 and S100A9, both independently and as a S100A8/A9 heterodimer [13,33].

Intracellularly, S100A8/A9 promotes phagocyte migration in a calcium dependent manner [24]. It is also associated with fibrosis and calcification [11]. Extracellularly, S100A8/A9 is primarily released from activated or necrotic neutrophils and monocytes/macrophages, and acts as an innate immune mediator in various inflammatory diseases [13,24] such as rheumatoid arthritis, bacterial infections, systemic lupus erythematosus, sepsis, and inflammatory bowel disease. It is also found in preeclamptic women [16,17,18]. S100A8/A9 may be the most informative serum analyte for identifying SLE, along with cognitive impairment [34], and is also significantly elevated in rheumatoid arthritis, colitis [35] or deep vein thrombosis [36]. Plasma S100A8/A9 has been found to be elevated in patients with septic shock, and their release leads to myocardial dysfunction [25].

At the site of infection, neutrophils eliminate the invading pathogens utilizing a combination of (nicotinamide adenine dinucleotide phosphate oxidase) NADPH oxidase-derived reactive oxygen species (ROS) with proangiogenic or immunosuppressive functions, with S100A8/A9 acting as an important neutrophil mediator [25,26]. Another mechanism by which the heterodimeric S100A8/A9 complex exerts antimicrobial effects is through its ion chelation properties. It has been established that S100A8 and S100A9 can chelate Zn^2+,^ Ca^2+^ and Mn^2+^, limiting the uptake of essential metal nutrients for pathogens. In this manner, calprotectin is implicated in resisting bacterial infection [27].

The S100A9 protein has been observed in cancers, where it is involved in invasion, migration, and the epithelial–mesenchymal transition (EMT) by promoting matrix metalloproteinase expression through the Wnt/beta-catenin and p38 MAPK-signaling pathways [37]. They contribute to angiogenesis, cell proliferation, and apoptosis [2,12]. In addition, a subtype of S100A8 and S100A9 + macrophages has been found to be associated with gastritic malignancy [38]. In lung adenocarcinoma, tumor-associated macrophage (TAM) infiltration significantly increases, causing the release of S100A9, which binds to the RAGE receptor on the surface of lung adenocarcinoma cells, and then activates the NF-κB pathway to promote EMT. This process leads to progression of the cancer [39].

## 4. Preeclampsia Physiopathology and Molecular Biology

### 4.1. Trophoblast Invasion, Uterine Spiral Artery Remodeling, and the Potential Role of S100A8, S100A9, and Calprotectin

Understanding the molecular and cellular pathways of endovascular invasion and the pathogenesis of pre-eclampsia are important topics for current vascular and placenta biology. The pathogenic mechanism of early-onset PE is complicated. It is related to shallow trophoblast invasion into the decidual layer and defective uterine spiral artery remodeling. After delivery, most women recover, and very few continue to suffer hypertension at 6 weeks after partum [40].

Adherence to the uterine wall of the embryo, which is a semi-allogenic graft, leads to invasion of the trophoblast in the decidualized endometrium during the “implantation window”. The embryo embeds itself, and extravillous trophoblast cells lose their polarity and enter the blood vessels. In the blood vessels, they lose their epithelial characteristics and communicate with maternal immune cells, NK cells, lymphocytes, and macrophages which produce cytokines that have a key role in the invasion of trophoblasts [3,7,41,42,43,44,45,46]. Pro-inflammatory macrophages also induce high level of Th17 cells at the maternal-fetal interface which leads to preeclampsia [47]. Baker et al. [48] presented evidence that S100A8, without S100A9, has a role in embryo development that was previously unrecognized. S100A8 also has a role in the maternal decidua, where its expression is associated with angiogenesis [2]. Calcium-binding proteins such as S100A9 have been studied in animal models, and the potential role of calcium and vitamin D has been observed in the establishment of pregnancy and the regulation of fetal and placental growth [40]. Calprotectin induces NLRP3 inflammasome activation through TLR4 pathway. S100A8/S100A9 bind to RAGE, release the pro -inflammatory cytokines, inhibit the matrix metalloproteinase that may lead to the dysfunction of uteroplacental circulation. Calprotectin may function in preeclampsia through interaction with RAGE and TLR4 receptors, [49,50,51]. which may lead to premature newborns with very low birth weight [52]. The presence of S100A8 and S100A9 was observed through immunohistochemistry for placental trophoblast cells, probably being a consequence of placental oxidative stress. Ca^2+^- binding proteins of the S100 family in preeclampsia [49].

High level of calprotectin has been found in endometrial tissue in women with endometriosis. In placental hypoxia a high amount of ROS is released and also antioxidants are reduced, such as superoxide dismutase or glutathione peroxidase.This process is associated with calprotectin release [49,53].

The trophoblasts’ invasion of vessels enables the release of immunoregulatory signals to immune cells at locations distant from the uterus, generating a systemic maternal immune response [46]. Inadequate signaling by the conceptus or a suboptimal immunologic maternal response can generate an unbalanced inflammatory reaction, which can lead to abnormal adaptation of the uterine vasculature [3,54].

Intra-arterial trophoblast plugs serve several important functions: protecting the embryo from toxic reactive oxygen metabolites and maintaining a low oxygen gradient [54,55,56]. Their dysfunction leads to PE, IUGR, and preterm delivery [57]. In 1967, it was first discovered that PE is associated with impaired trophoblast invasion and impaired remodeling of spiral artery walls with placental malperfusion [7]. Maternal overexpression of S100A8/S100A9 increases the recruitment of inflammatory leukocytes in placenta resulting in uteroplacental mal-perfusion and development of thrombotic events, causing hypoxia, which may lead to miscarriage [58,59]. Decidualization and the formation of an “implantation window” are carried out under the influence of estrogens and progestogens [36,46,51]. In PE, unbalanced vaso-activity is encountered [51,52,54]. It has been shown that imbalanced remodeling of the spiral arteries in the uterus leads to placental ischemia/hypoxia [2,60]. S100A8 is sensitive to oxygen. Low-oxygen conditions prevail during the early stages of trophoblast invasion, and hypoxic conditions induce cell stress and S100 protein release [61,62]. S100A9 seems to be less susceptible to oxidation compared to S100A8 [13,30]. The hypothesis that S100A8/A9 oxidation releases S100A9 under mild oxidative conditions would explain the lack of receptor TLR4 and RAGE activation under physiological conditions. This issue remains controversial, and it needs further clarification [13].

**Figure 2:**
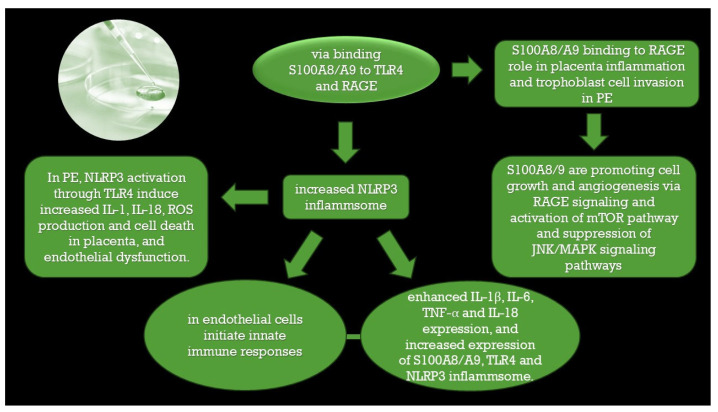
Calprotectin (S100A8/S100A9) is acting after binding to TLR4 and RAGE receptors.

It has been already demonstrated that S100A8/S100A9 is released in ischemia/reperfusion injury [13]. The placenta is a rich source of reactive oxygen species (ROS), reactive nitrogen species (RNS), and lipid peroxides which alter cytokine expression. In PE, a high level of ROS changes the balance in expression/levels in favor of antiangiogenic factors. This may result in damage to vascular endothelial cells [2]. Placental ischemia/hypoxia may lead to an elevated level of circulating calprotectin (S100A8/S100A9 heterodimer), S100A8, and S100A9, which may be future biomarkers of endothelial damage during pregnancy [59]. Neutrophil activation during uteroplacental circulation passage is the leading cause of this. This process causes increased expression of IL-6 (interleukin 6) and TNFα (tumor necrosis factor α) [2,62], along with neutrophil activation [63] and calprotectin release [2,64]. Some of these mechanisms are presented in the figure below (Figure 2).

Increased calprotectin in serum inhibits the activity of matrix metalloproteinases (MMPs); matrix metalloproteinases are zinc-dependent enzymes, while calprotectin acts as a zinc scavenger. MMPs play key roles in trophoblast cell invasion. Dysregulation of MMPs might contribute to reduced trophoblast invasion [2,64].

S100A8 and S100A9 are gaining increased attention as inflammatory markers for PE [18]. Systemic inflammation and leucocyte activation have long been established as pathological causes of PE, and because S100A8 and S100A9 are secreted by neutrophils, they are involved in promoting the inflammatory response in PE [18]. S100A8, S100A9, and S100A8/A9 promote neutrophil and monocyte recruitment and cytokine secretion. The mechanism seems to be the activation of the microvascular endothelium and the stimulation of Mac-1 expression, binding to ICAM and fibrinogen [13,19,20,21,65]. S100A8/A9 is also locally released in ischemia/reperfusion injury [13]. Endothelial activation during pregnancy is characterized by higher levels of coagulation in endothelial markers and matrix-metalloproteinases (MMPs). In pregnancy, vascular remodeling is associated with changes in extracellular matrix composition and MMP activity. MMP-2 processes and modulates the functions of S100A8 and S100A9. These molecules could become potential molecular markers of endothelial damage during pregnancy [66].

Another hypothetical PE etiology is inflammation of noninfectious origin, caused by endogenous mediators known as alarmins or “damage-associated molecular patterns (DAMPs)” [3,12,13]. Classic examples of DAMPs investigated in this review are uric acid, cell-free fetal deoxyribonucleic acid (DNA) (cfDNA), mobility group box 1 (HMGB1), adenosine triphosphate (ATP), and heat shock protein 70 (HSP70) and S100 proteins [67,68]. The main placental cells, such as trophoblast and Hofbauer cells, are involved in the placental response to DAMPs, with a role in pregnancy complications including PE, IUGR, preterm birth, and stillbirth [67,68]. Hofbauer cells, which are fetal cells implicated in the vascular remodeling process, are a poorly described population of fetal macrophages within the stroma of the healthy placenta. HBCs secrete factors, such as osteopontin, galectin, and MMP-9, with an effect on angiogenesis and vessel remodeling [67]. Damage-associated molecular patterns (DAMPs) are downregulated by Hofbauer cells, with implications in pregnancy complications such as PE and IUGR with smaller placentas and impaired spiral artery remodeling with fetal growth restriction [68,69]. Researchers have proposed oxidant scavenging as another potential anti-inflammatory mechanism for S100A8, S100A9 and S100A8/A9 [70], along with Zn^2+^ chelation with an inhibitory effect on matrix metalloproteinase (MMP), or inhibition of ROS production by phagocytes [2,13]. These processes, along with chemotactic activity for myeloid cells and recruiting immune cells such as NK cells into maternal tissue, could be involved in PE [48].

### 4.2. Potential Clinical Significance of S100A8, S100A9, or Calprotectin in PE

PE remains a leading cause of fetal and maternal death worldwide. PE is associated with substantial health risks during and after pregnancy, potential to affect the lives of those with the diagnosis of preeclampsia and their children [71]. In this review, we highlight various pathophysiological mechanisms involved in PE that have been described in other reviews and original articles, as well as describe the possible importance of S100A8, S100A9 and the heterodimer S100A8/S100A9 in these mechanisms. We intended to emphasize that PE is a multifactorial disorder [54].

Recent research strongly emphasizes that the quality of implantation determines the quality of the ongoing pregnancy. The crosstalk between the conceptus and the mother begins before implantation [48,54,67]. The inflammasome NLRP3 NOD-like receptors’ protein 3 is involved in PE [57]. In an animal study, pregnant mice were administered S100A9. S100A9 activates the NLRP3 inflammasome in mice trophoblasts. Secretion of soluble endoglin is regulated via S100A9-stimulated NLRP3 inflammasome activation. S100A9 administration significantly increased neutrophil activation within the placenta of pregnant mice, which in turn led to a significant increase in blood pressure [11,72].

Calprotectin is two times higher in the serum of women with PE; its levels significantly increase in the third trimester, and it can trigger an inflammatory reaction. The PE placenta is characterized by ischemia and infarction; this hypoxic environment leads to increased IL-6 and TNF alfa expression and causes neutrophil activation with cytokine and calprotectin release [2].

Furthermore, the upregulation of S100A8 and MMP8 have been observed in association with adverse pregnancy outcomes (APOs) [73]. Elevated levels of high mobility group protein B1 (HMG-1) and calprotectin were measured among patients with PE compared to normal pregnant women by Jinfeng Li et al. (2018) [74]. Their levels were positively associated with the duration of hypertension in pregnancy, which reflects an excessive systemic inflammatory response in PE [74].

Xiaoyun Li et al. (2019) compared the expression of calcium-binding protein S100A8 using S100A8 mRNA detected via reverse transcription polymerase chain reaction (RTPCR) in 60 women with PE as an experimental group and 30 normal pregnant women as a control group [75]. They found a statistically relevant high level of S100A8 in the experimental group at the S100A8 mRNA and protein level. They also found a positive correlation between S100A8 protein levels and the levels of uric acid and urinary proteins [75]. Calprotectin levels were elevated in the amniotic fluid in pre-eclamptic pregnancies [76,77].

S100A9 protein has been observed in cancers [37]. Starting from the similarities between cancer and pregnancy, researchers have studied the role of S100A9 in early pregnancy, and found that upregulated S100A9 in early pregnancy mediates trophoblast function [37]. Pregnancy has features that are similar to those of cancer, but its development is limited in space and time [3]. Successful placentation requires communication between the trophoblast and endometrium, mediated by the hormonal environment, which modulates trophoblast function. Estrogen and progesterone mediate S100A9 secretions in the endometrium. This process enhances trophoblast invasion during early pregnancy [37]. Dysregulation of these functions leads to various pregnancy complications such as miscarriage and PE. In one study, Malique et al. (2017) the researchers showed that S100A9 facilitates invasion, migration, and the epithelia–mesenchymal transition (EMT) in the early phase of pregnancy and may also be involved in the remodeling process of spiral arteries during pregnancy, depending on its level [37].

It is well known that the S100A8/S100A9 heterodimer (calprotectin), through interaction with endogenous Toll-like receptor 4 (TLR-4), is involved in immune responses, cell proliferation, and apoptosis [2,78]. The calprotectin level is twice as high in the serum of pre-eclamptic women in comparison to healthy pregnant patients [79]. This level gradually increases with time and disease severity and is positively correlated with the duration of hypertension [74]. Bushra Iftikhars and Aslam Alya (2020) found a statistically significant increase in calprotectin levels in correlation with gestational age [18].

Brien et al. [80] evaluated 30 mediators in plasma samples, including cytokines and alarmins, obtained from the late second trimester and at delivery from 100 pregnant women with PE/IUGR (50 patients each) and compared these to 100 normotensive pregnant women. They found increased levels of inflammatory mediators (ex. IL-6) compared to the control, supporting an inflammatory profile in PE [80]. In PE, abnormal neutrophil activation occurs, and the endothelium releases reactive oxygen species (ROS) into the maternal circulation; these species can promote a systemic inflammatory response [81].

**Figure 3:**
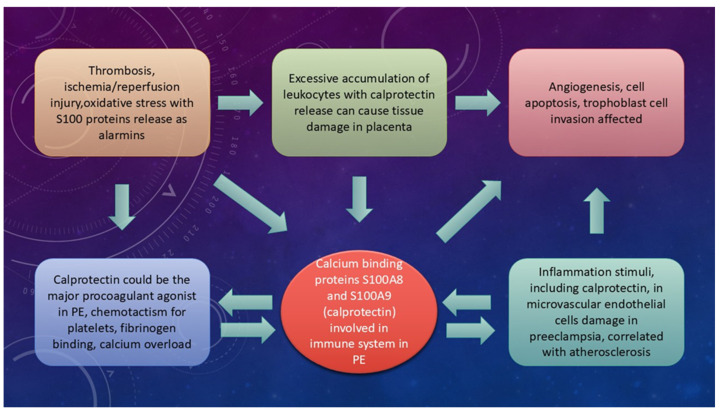
The connection of S100A8 and S100A9 with preeclampsia.

A possible connection of S100A8 and S100A9 with pathogenesis of the placenta, through vascular damage, is presented in Figure 3.

### 4.3. Different Pathologies as a Risk Factor for PE and the Association of S100A8 and S100A9

The recognized risk factors for PE, according to the American College of Obstetricians and Gynecologists (ACOG), include nulliparity, a history of PE in a previous pregnancy, chronic hypertension, pre-gestational diabetes, gestational diabetes, multiple gestations, thrombophilia, antiphospholipid syndrome, systemic lupus erythematosus, pre-pregnancy body mass index (BMI) greater than 30, maternal age of 35 years or older, assisted reproductive technology, kidney disease, and obstructive sleep apnea [7,82]. The risk factors for cardiovascular disease, as identified by the American Heart Association [7] and the American Stroke Association [83], overlap with those for PE, except for factors specific to pregnancy. Giannakou et al. [84] emphasize that both share many risk factors [7]. In pregnancy, the maternal cardiovascular system is under stress, and when it is in a poor condition, complications become inevitable [7]. In those women with PE, cardiovascular complications after pregnancy may occur [7]. Due to hemodynamic changes, an adequate uteroplacental blood flow is ensured; this is necessary for the growing fetus [7,83].

Primary pathologies of the vessel walls can lead to placental malperfusion. Some autoimmune disorders, such as lupus or Sjogren, together with diabetes with enlarged placentas, antiphospholipid syndrome, and inherited or acquired thrombophilia, can serve as a possible cause for impaired placental remodeling and malperfusion [3,7,54,85]. Antiphospholipid syndrome (APS) is an autoimmune disorder associated with several obstetrical complications such as PE, IUGR, and fetal death [2,3,8,9].

Serum and plasma levels of calprotectin are elevated in inflammatory diseases, such as lupus erythematosus, rheumatoid arthritis RA, and inflammatory bowel disease, and in thrombotic diseases such as myocardial infarction and COVID-19 [40,71,84,86,87]. S100A8/A9 is an active mediator in the pathogenesis of various autoimmune and inflammatory conditions [13]. Patients with SLE and antiphospholipid antibodies have increased levels of platelet calprotectin and fecal calprotectin. Proinflammatory factors are needed in this process. Calprotectin may be an independent risk factor for microvascular manifestations. It has also been associated with thrombocytopenia in those patients; this condition occurs in 20–50% of aPL-positive patients. The mechanism is unclear [3,40]. The risk of CVD is elevated in patients with autoimmune rheumatic diseases, and S100A8/A9 is also high in SLE patients with CVD [10]. S100A8/A9 binds heparan sulphate proteoglycans [88,89] triggering endothelial activation, with enhances production of inflammatory cytokines and chemokines [13,19], increases platelet aggregation at the endothelium [25], and causes high expression of adhesion molecules [19]. This complex process leads to endothelial cell dysfunction and increased endothelial permeability. S100A8/A9 have also been shown to downregulate the genes responsible for the integrity of the endovascular monolayer in endothelial cells [19,89].

Pregnancies affected by diabetes mellitus are at risk for PE. Excessive neutrophil activation occurs in cases with PE, leading to the presence of many neutrophils in the placenta. This activation also exhibits similar behavior in women with gestational diabetes mellitus associated with PE. Neutrophil activity in gestational diabetes mellitus may contribute to the development of PE, as this activation is involved in inflammation, oxidative stress, and defective angiogenesis [89,90]. Satish P. Ramachandrarao (2016) evaluated the exosome proteome content from 24 h urine samples of 18 pregnant subjects with gestational diabetes mellitus and compared the results with control samples of 10 subjects obtained at week 20 of pregnancy [91]. S100 calcium-binding protein A9 was found to be significantly increased in diabetes mellitus. Exosome biomarkers could potentially be used in women with diabetes with the aim of improving pregnancy outcomes [91].

Diabetes mellitus, hyperlipidemia, obesity and smoking are traditional CV risk factors. They also have been associated with increased levels of S100A8/A9 in plasma. Hyperglycemia induces the production ROS in human endothelial cells with increased endothelial permeability in animal models, with the overexpression of S100A8 and RAGE [29]. Similarly, increased S100A8/A9 secretion in neutrophils could be induced by hyperglycemia [13,40,81]. Body mass index (BMI) has been independently linked to S100A8/A9 plasma concentrations [92], and significantly decreased S100A8/A9 is associated with weight loss [92]. Urinary exosomes containing S100 calcium-binding protein A9 were found to be significantly increased in pregnant women with diabetes mellitus, with high risk for developing PE. It is also independently correlated with maternal obesity [91]. Exosomes containing S100A9 were found in women with endothelial injury and PE [11].

Pregnancy is a trigger factor for the development of congenital thrombotic thrombocytopenic purpura (TTP), with an increased risk of maternal and fetal complications, including PE, IUGR, and fetal demise. There is microscopic evidence of fetal and maternal vascular lesions of under-perfusion in their placentas, intra-placental infarcts, fibrin thrombi, and intervillous fibrin depositions [10,92,93,94,95]. Platelets have a proinflammatory role. Platelet–neutrophil interaction has been reported in thrombotic conditions. S100A8/A9 binds to TLR4 Toll-like receptor 4 expressed on platelets. Platelet hypercoagulability was found in situations of high serum levels of calprotectin, which increased platelet–neutrophil activation [84,85,86,87,88,89,90,91,92,93,94,95,96,97,98,99,100,101]. Oxidative modifications of S100A8 and S100A9 induced by ROS mainly target cysteine and methionine residues promoting endothelial lesions and coagulation [13]. Confirmation of congenital or immune subtypes is essential in the management of subsequent pregnancies. In the case of TTP with fetal demise, proteomics analysis identified a 6-fold to 7-fold overexpression of S100A8 and S100A9 [96]. Complement-mediated hemolytic uremic syndrome (CM-HUS) is another microvascular disease that can appear at any time, from the early stage of pregnancy to the postpartum period. CM-HUS may be further complicated by PE and HELLP, which are specific thrombotic microangiopathies in pregnancy, present from the second trimester [96,97]. The micro-thrombosis found in COVID-19 is also a thrombotic micro-angiopathy that can be complicated by PE, with high level of calprotectin in plasma. Ultra-large von Willebrand factor multimeric glycoproteins are released through the endothelium into the blood stream, and the ADAMTS13 is insufficient to cleave the large vWF multimers, thus activating intravascular thrombosis [98].

When platelet–neutrophil aggregates are found in the microvasculature during inflammation, endothelial injury occurs, with high ROS production and placental damage [10,93,99]. Platelet activation contributes to the pathophysiology of PE and plays a key role in the prothrombotic state. Platelets in PE are partially degranulated and can circulate as microaggregates/microthrombi. This dynamic reveals novel drug targets and a potentially suitable alternative to aspirin for the management of prothrombotic tendencies in PE [100].

Platelets are key regulators of thrombotic complications in PE, linking inflammation and thrombosis with endothelial dysfunction. Fibronectin and S100A8/9 may be major procoagulant agonists in PE, and this process is probably fibrinogen-driven. Absolute changes have been observed in S100A9, cadherin-5, caspase-12, fibronectin, and apolipoprotein. S100A8/9 has been previously associated with a predisposition for cardiovascular diseases, metabolic inflammation, and changes in platelet activity. However, elevated levels of S100A9 in lysates of pre-eclampsia platelets have been identified [1]. S100A8/A9-positive neutrophils and macrophages infiltrate the occluding thrombus, and S100A9 mRNA transcripts are increased in circulating platelets [12]. A causal role for S100A9 in thrombosis has been reported, with micro-aggregation and clumping in a concentration-dependent manner [1,101]. However, the presence of S100A9 mRNA in platelets has been debated by other researchers, as platelets in the occluding thrombus were not observed to express the S100A8/A9 protein in other studies [13,101,102,103].

The placenta acts as the primary driver of cardiovascular dysfunctions in PE. This observation is supported by the disappearance of PE symptoms after delivery [57]. Signs of atherosclerosis in uterine spiral arteries have been reported [7,57]. Foam cells are also present in the arterial walls of the decidua, a characteristic of acute atherosis, probably as a reaction to inflammatory triggers [7,44,103]. There is a pathophysiological connection between the two stages of PE. The first stage is characterized by placental malperfusion, and the second stage is represented by maternal endothelial dysfunction. This connection consists of the increased production of antiangiogenic factors by poorly perfused placentas. These women later develop PE because of maternal endothelial damage through these antiangiogenic factors [98,104]. Vasoconstriction and platelet aggregation occur in systemic vascularization as well [3,105]. Targeted screening evaluation should be established to prevent potential consequences for heart health, and obstetricians must explain the elevated risk of cardiovascular events in these women compared to the general population [7]. The link between S100A8/A9 and atherosclerosis is supported by clinical studies [12,13].

## 5. The Clinical Relevance of S100A8/A9 in Intrauterine Growth Restriction (IUGR)

Pre-eclampsia is one of the leading causes of IUGR, which remains a significant concern in obstetrics and is linked to neonatal death, severe health problems, and childhood disability [76]. Compared with the American College of Obstetricians and Gynecologists’ definition of PE, a more inclusive definition was proposed by the International Society for the Study of Hypertension in Pregnancy which included maternal end-organ dysfunction, uteroplacental dysfunction, and the imbalance of angiogenic factors. IUGR is one of the most important features of PE [5,71]. IUGR is common in placental insufficiency and inflammation. The expression of S100A9 is known to be involved in this process [40,71], and NLRP3 may also contribute [76].

IUGR is associated with the activation of complement and coagulation cascades. A significant increase in proinflammatory S100 calcium-binding protein A9 was found in early-onset PE/IUGR. Incorrect placental development with local ischemia have been associated with the release of S100A8/A9 protein [71,76,106,107,108].

Non-invasive prenatal testing (NIPT) is a commonly used method for understanding fetal health. It is frequently used because researchers focus on fetal health rather than maternal health. However, blood has also been analyzed to evaluate maternal health, and S100A9 was elevated in PE and strongly associated with inflammation, which is common in PE. There is a positive significant correlation between the level of calprotectin and the blood pressure [76,105]. Parathormone (PTH) and calprotectin play a substantial role in IUGR. Calprotectin is higher in the amniotic fluid in IUGR fetuses compared with normal fetuses due to oxidative stress [71,72,109,110]. This increase is also important because an adverse intrauterine environment, due to conditions such as gestational hypoxia and fetal stress, causes epigenetic reprogramming of methylome and transcriptome in the development of the fetal heart, leading to an increased risk of fetal heart disease later in life [111].

Analysis of maternal plasma cell-free content has been an interesting option for screening genetic abnormalities during pregnancy. Less attention has been paid to adverse pregnancy outcomes (APOs) based on placental dysfunction. For example, placental-specific DNA increases prior to the subsequent development of gestational diabetes, and an upregulation of S100A8 has been associated with APOs [111].

Maroudias et al. collected amniotic fluid samples from pregnancies at 15 to 22 weeks of gestation. In total, they collected samples from 64 pregnancies and measured calprotectin values in the amniotic fluid. In the early second trimester, calprotectin levels were higher in fetuses that were large for their gestational age compared to those in fetuses that were small or normal for their gestational age. This situation can possibly be explained by oxidative stress and, eventually, the release of calprotectin caused by low-grade chronic inflammation due to excessive fat deposition [77]. Ackum et al. (2010) explored the plasma level of calprotectin in preeclamptic women versus normal pregnancy and non-pregnant women, with significant increase in calprotectin in preeclamptic group, but the small sample size of patients decreases the relevance of data reported [112].

The importance of S100 proteins in PE is summarized in the table below (Table 1).

## 6. Relevance in Future Therapy

Currently, the first-line therapy for PE is antihypertensive drugs. In order to reduce the risk of developing PE, low doses of aspirin should be initiated early in high-risk pregnancies. In 2013, the ACOG published a report which supported the use of low-dose aspirin in pregnant women with a previous history of early-onset preeclampsia [71].

The calcium-binding protein S100A8 can exert anti-inflammatory functions by scavenging ROS and reducing oxidative stress. IL-35 inhibits endothelial dysfunction directly, and IL-3 upregulates S100A8 expression in HUVECs in vitro experiments [113]. In addition, as we know that TLR 4, the receptor for S100A9, facilitates the upregulation of proinflammatory cytokines, TLR4 could be a novel therapeutic target [114,115,116]. Quinoline-3-carboxamides are chemical compounds with anti-inflammatory properties in various autoimmune diseases such as SLE, arthritis, experimental autoimmune encephalomyelitis. Phase II and III treatment are under development for anti-inflammatory/autoimmune disease [114,115]. Recently, Björk at al. have identified the elusive target of quinoline-3-carboxamides which is S100A9 [114]. Quinoline-3-carboxamide binds S100A9 and S100A8/A9 in a Ca^2+^- and Zn^2+^-dependent manner in animal and human models, blocking their interaction with RAGE and TLR4 [114].

Following ischemia, S100A8/A9 is released in circulation, mainly from activated neutrophils and monocytes/macrophages. Administering S100A8/A9 blockade inhibits systemic inflammation, reduces ischemic injury, and enhances angiogenesis in myocardial dysfunction in order to increase nutrient and oxygen delivery in the ischemic area in animal model [26]. It would be interesting to evaluate if the same process could be obtained in preeclamptic placenta. Last but not least, S100A8/9 may be targeted for antithrombotic treatment and can be used as a biomarker in PE [33].

Encouraging results have been obtained in experimental and clinical interventional studies with S100A8/A9 blockers on autoimmune disease and cancer [13].

Prospective studies are needed to determine whether S100A8/A9 measurement in placenta and plasma can offer independent information for preeclampsia. The main obstacle is the relative abundance of this protein in human circulation. Women with intra-amniotic infection were found to have a significantly higher intra-amniotic calprotectin concentration compared to women with normal pregnancy [63,117]. Neither the clinical options of A100A8/A9 proteins nor their biomarker potential are evident at this time; future investigations are required.

As a limitation of our review, in the cited studies calprotectin importance in preeclampsia is not demonstrated only correlations being reported. A systematic review should be appropriate that would more explicitly distinguish between associations and proven causal mechanisms. This is why the integration of calprotectin measurements into non-invasive prenatal testing frameworks is not yet appropriate. Another limitation is that the calprotectin inhibitors presented as future treatment are in various experimental phases of treatment in humans. But administration during pregnancy is problematic due to safety issues for the fetus.

Additionally, more research is needed to explore the mechanisms behind the various causes of pre-eclampsia. It seems that proper management of this condition should be carried out with a multidisciplinary approach and using variations in treatment that should be personalized to each patient.

## 7. Conclusions

Pre-eclampsia is a common pregnancy complication that may threaten the health of pregnant women and their fetuses. Multiple biomarkers usually increase successful diagnosis of this condition.

Even if encouraging results have been obtained with S100A8/A9 blockers for treating autoimmune disease and cancer, their use in preeclampsia has not yet been validated. It may also be limited because of safety issues for the fetus.

Understanding the pathological mechanisms and the importance of calprotectin could shape a new strategy for large-scale approaches to changing the diagnostic and therapeutic management of this condition. However, future investigation can emerge, based on our review, such as to investigate the predictive value of combining S100A8/A9 with established biomarkers (sFlt-1/PlGF) and the potential of S100A8/A9 as a therapeutic target in PE-related thrombosis.

## Figures and Tables

**Table 1:** The correlation between S100A8, S100A9, Calprotectin, and preeclampsia.

Research	S100A8, S100A9, Heterodimer S100A8/S100A9 or Calprotectin	Preeclampsia	IUGR	Influence	Sample Size	Signifficance
de Almeida (2022) [1]	S100 as proteomics	yes	yes	PE in relation with cardio-vascular diseases; contribute to angiogenesis, apoptosis and proliferation	17	Small sample size, not signifficant
Jurewicz, Ewelina (2022) [2]	S100	yes	Including IUGR	Inflammation	Review	Detailed
Singh, Parul (2022) [10]	S100	yes	yes	Affecting Immune system	Review	Few elements regarding calprotectin
Cotoi, Ovidiu (2014) [12]	S100A8, S100A9, heterodimer S100A8/S100A9 or calprotectin	yes	NA	PE in relation to cardio-vascular diseases; correlates with atherosclerosis, including in placenta, in ischemia/reperfusion injury	664	Large sample but related to cardio-vascular disease, few details about PE
ASLAM, ALIYA (2020) [18]	calprotectin	yes	NA	Serum calprotectin as inflammatory biomarker in PE	24	Article from a grey zone
Viemann D (2005) [19]	S100A8	yes	NA	Inflammation of endothelial cells	There is no sample size declared	Inconsistent data
Stenhouse, Claire (2021) [40]	S100A8, S100A9, heterodimer S100A8/S100A9 or calprotectin	yes	NA	establishment of pregnancy and the regulation of fetal and placental growth, faecal calprotectin as biomarker	Animal study	Good quality
Zhao, Yuan (2023) [65]	calprotectin	Yes in antiphospholipid syndrome	NA	Serum calprotectin in microvascular manifestations	466	Relevant information
Maliqueo, M (2016) [37]	S100A9	yes	NA	trophoblast invasion during early pregnancy, artery remodelling	review	Few information regarding calprotectin
Lai, J. (2021) [5]	calprotectin	yes	NA	Serum level of calprotectin as biomarkers for PE	In vitro experiment	Relevant information
Xiaoyun Li (2019) [75]	S100A8	yes	NA	Urinary S100A8 as biomarker in PE	90	Relevant information, good quality research
Braekke, Kristin (2005) [64]	Calprotectin	yes	NA	Serum calprotectin marker of inflammation in PE, but not in their offsprings	69	Relevant for our review
Brien, Marie-Eve (2019) [68]	S100A8, S100A9, heterodimer S100A8/S100A9 or calprotectin as DAMPs	yes	yes	trophoblast and Hofbauer cells, are involved in the placental response to DAMPs, with role in pregnancy complications including PE, IUGR,	review	Relevant information
Gomes L. H. (2013) [70]	S100A8 and S100A9	yes	yes	as oxidant scavenging in inflammations	56	Few relevant information for PE
SP Ramachandrarao (2016) [91]	S100A9	yes	yes	Diabetes Mellitus, urinary exosomes as biomarkers in association with PE	28	Small sample size
Robinson M. J (2002) [89]	S100A8/A9	yes	yes	S100A8/A9 binds heparan sulphate proteoglycans, coagulation cascade	In vitro experiment	High quality study design
Ortega F. J (2012) [92]	calprotectina	yes	yes	Serum and urinary calprotectina related to inflammation in Diabetes Mellitus and PE	298	Few link to PE
Skeith, Leslie (2022) [96]	S100A8 and S100A9	yes	NA	TTP with fetal demise, proteomics analysis identified a 6-fold to 7-fold overexpression of S100A8 and S100A9	Case report	Can not be generalized
Sureshchandra S (2021) [101]	S100A9	yes	NA	A causal role for S100A9 in thrombosis has been reported, with micro-aggregation and clumping	15	Small sample size with no control group identified
Li J (2018) [74]	S100A9	yes	yes	Common in placental insufficiency	Special report	Clear information and recommendationes
Jencks (2024) [110]	calprotectina	yes	yes	Serum calprotectin as biomarker in colitis, determin IUGR	report	Few information
Maroudias, George (2024) [77]	calprotectin	yes	yes	Higher level of calprotectin in amniotic fluid in PE with IUGR, caused by oxidative stress	64	Relevant for our review
Li, Ming (2020) [113]	S100A8	yes	yes	Upregulated by IL-35, in PE targeted treatment	48	Relevant information
Björk P. (2009) [114]	S100A9 as target	PE in autoimmune diseases such as SLE	yes	Targeted treatment with Quinoline-3-carboxamides	In vitro experiment	Relevant information
Bengtsson A. A (2012) [115]	S100A9 as target	PE in autoimmune diseases such as SLE	yes	Targeted treatment with Quinoline-3-carboxamides	Animal model	Relevant information
Ackum(2010) [112]	Serum Calprotectin	PE	yes	Biomarker	Small sample size	Low relevance

PE—preeclampsia, IUGR—intrauterine growth restriction, DAMPs—damage-associated molecular patterns, TTP—thrombotic thrombocytopenic purpura, SLE—systemic lupus erythematosus, NA-not aplicable

## Data Availability

All relevant information is in corresponding author possession.

## Abbreviations

PE	Preeclampsia
HELLP	syndrome hemolysis, elevated liver enzymes, low platelet count
IUGR	intrauterine growth restriction
ROS	reactive oxygen species
sFlt1	soluble FMS-like tyrosine kinase 1
s Eng	soluble endoglin
PAPP-A	pregnancy associated placental protein- A
PlGF	placental growth factor
TLR4	toll-like receptor 4
RAGE	the receptor for advanced glycation end products
DAMPs	danger-associated molecular patterns
SLE	systemic lupus erythematosus
NADPH	nicotinamide adenine dinucleotide phosphate oxidase
EMT	epithelial–mesenchymal transition
TAM	tumor-associated macrophage
NLRP3	inflammasome NOD-, LRR- and pyrin domain-containing protein 3 inflammasome
RNS	reactive nitrogen species
MMPs	matrix metalloproteinases
DNA	deoxyribonucleic acid
HMGB1	mobility group box 1
ATP	adenosine triphosphate
HSP70	heat shock protein 70
IL	interleukin
TNF	tumor necrosis factor
ACOG	American College of Obstetricians and Gynecologists
BMI	body mass index
aPL	antiphospholipid antibodies
CVD	cardio-vascular disease
TTP	thrombotic thrombocytopenic purpura
CM-HUS	Complement-mediated hemolytic uremic syndrome
vWF	Ultra-large von Willebrand factor multimeric glycoproteins
NIPT	Non-invasive prenatal testing
APOs	adverse pregnancy outcomes
HUVECs	human umbilical vein endothelial cells
